# Supplementary material for: Transcriptomic and Proteomic Analysis of Gardnerella vaginalis Responding to Acidic pH and Hydrogen Peroxide Stress
Source: Microorganisms. 2023 Mar 8;11(3):695. doi: 10.3390/microorganisms11030695 (PMC10051395; doi:10.3390/microorganisms11030695)

Table S1. Primers for QPCR.

GeneID	Function	Sequence (5'-3')
HMPREF0421_20131	subunit A of gyrase	F: TGACACTTTGGTTCGTATGGC<br>R: TCCGTATAACGCATTGCTGC
HMPREF0421_20660	sensor histidine kinase	F: GTGGTACTGGCTTAGGTATGTCT<br>R: ATCACTGCTGGTCCTAGATGC
HMPREF0421_20990	sensor histidine kinase	F: CGAAATGGCTGAAGAACTAGAGC<br>R: TAGCTTGTAATGCACTAACAGGTG
HMPREF0421_20118	thioredoxin-disulfide reductase	F: GCAAGAATACTCTGGACGAGGT<br>R: CAAAAGCTGAATCGCCTCCG
HMPREF0421_21330	glutaredoxin	F: GCGTCAATTCACTAAGCTAGGC<br>R: GAGTAATCACCACTGGCGCT
HMPREF0421_21015	3-oxoacyl-ACP reductase	F: ACGCAACAAACCATGCTTTAT<br>R: GCGTATGCTGCTGTTCCTAG
HMPREF0421_21022	sensor histidine kinase	F: GCCTTATTGTTGCAGCAATAGC<br>R: CCTTTGGATCCCATTGCTGC
HMPREF0421_21221	sulfonate ABC transporter permease	F: TATTCGTCATTCGCATGCCG<br>R: GTGAACCTGTGCGTCCGAAT
HMPREF0421_20219	major facilitator superfamily (MFS) transporter	F: ACCAGCAAGCACAAAATAGC<br>R: ACTCCTAAGACTATTAACCATGGTGT
HMPREF0421_20303	actinobacterial surface-anchored domain protein	F: CGAAAGAAACTAAAACCACTAATACTG<br>R: TCCGCGTTCTTTAACAACAGAC
HMPREF0421_20991	transferring glycosyl groups	F: TATTCGTGCTCAACTTATGTAGG<br>R: CCAAATATGAAAAAGGCTTATCG
HMPREF0421_20208	hydroxyethylthiazole kinase	F: GCAGAAGAAGCTGAAGTCTTTGC<br>R: CGAATCGCAGCATCCATAGATTC
HMPREF0421_21089	type IV prepilin peptidase	F: TGTGCCTAGACTTTGGGTTGT<br>R: TACTGCAAAAAGCCAGCCAC

Table S2. Unique genes in the Venn analysis.

Unique genes	GeneID	Function
Control	HMPREF0421_20665	integral component of membrane
	HMPREF0421_20284	hypothetical protein
	HMPREF0421_20681	hypothetical protein
	HMPREF0421_20017	hypothetical protein
	HMPREF0421_20419	hypothetical protein
	HMPREF0421_21040	inclusion membrane protein F
	HMPREF0421_21316	hypothetical protein
Lac	HMPREF0421_20047	hypothetical protein
	HMPREF0421_21359	type I restriction enzyme, R subunit
	HMPREF0421_20088	hypothetical protein
LacHyd	HMPREF0421_20371	CDP-diacylglycerol-glycerol-3-phosphate 3-phosphatidyltransferase

Table S3. List of the genes with fold changes above two among the differentially expressed genes in *G. vaginalis* cultured under lactate. All *P* values were less than 0.02.

GeneID	Log2Fold Change	*P* value	*P* adj	Function
Upregulated				
HMPREF0421_21022	3.65	5.42×10^{-166}	1.51×10^{-164}	Sensor histidine kinase
HMPREF0421_21024	3.24	1.31×10^{-18}	4.20×10^{-18}	ABC transporter
HMPREF0421_20927	3.15	0	0	ABC transporter substrate-binding protein
HMPREF0421_20219	2.97	4.12×10^{-71}	4.02×10^{-70}	major facilitator superfamily (MFS) transporter
HMPREF0421_21023	2.96	1.88×10^{-14}	5.23×10^{-14}	Hypothetical protein
HMPREF0421_21015	2.90	1.27×10^{-91}	1.64×10^{-90}	3-oxoacyl-ACP reductase
HMPREF0421_21026	2.73	5.07×10^{-21}	1.72×10^{-20}	ABC transporter ATP-binding protein
HMPREF0421_21025	2.70	6.04×10^{-33}	2.94×10^{-32}	ABC transporter ATP-binding protein
HMPREF0421_20844	2.59	0	0	Cobalt ABC transporter
HMPREF0421_20843	2.36	5.32×10^{-206}	2.16×10^{-204}	Membrane protein
HMPREF0421_20839	2.20	0	0	NADH dehydrogenase
HMPREF0421_20940	2.17	0	0	oligoribonuclease
HMPREF0421_21014	2.05	1.04×10^{-120}	2.04×10^{-119}	2-keto-3-deoxygluconate permease
Downregulated				
HMPREF0421_20991	-3.24	7.95×10^{-28}	3.39×10^{-27}	Transferring glycosyl groups
HMPREF0421_21002	-2.86	9.73×10^{-82}	1.06×10^{-80}	Cell division protein FtsK
HMPREF0421_20208	-2.75	0	0	Hydroxyethylthiazole kinase
HMPREF0421_21089	-2.51	4.12×10^{-12}	1.06×10^{-11}	Type IV prepilin peptidase
Novel C3	-2.46	9.40×10^{-20}	3.08×10^{-19}	Transcription regulator
HMPREF0421_20331	-2.24	5.36×10^{-58}	4.22×10^{-57}	Serine/threonine protein kinase
HMPREF0421_20303	-2.21	8.22×10^{-223}	4.08×10^{-221}	Actinobacterial surface-anchored domain protein
HMPREF0421_20304	-2.04	1.31×10^{-77}	1.38×10^{-76}	Hypothetical protein

Table S4. List of the differentially expressed genes in the Lac group compared to the LacHyd group. All *P* values were less than 0.02.

Gene ID	Fold change	*P* value	*P* adj	Function
HMPREF0421_20956	1.42	7.96×10^{-156}	3.55×10^{-153}	Trimeric autotransporter adhesin
HMPREF0421_20499	-1.37	8.86×10^{-11}	7.95×10^{-10}	LPXTG-motif cell wall anchor domain protein
HMPREF0421_20990	-1.17	1.35×10^{-33}	5.84×10^{-32}	Sensor histidine kinase
HMPREF0421_20489	-1.26	7.72×10^{-200}	1.03×10^{-196}	ABC transporter ATP-binding protein
HMPREF0421_20488	-1.09	1.28×10^{-177}	8.58×10^{-175}	ABC transporter substrate-binding protein

Figure S1. The expressional level of response markers after adding lactate and H_2O_2. Bars represent the mean and the error bars the standard error of the mean (mean ± SEM).

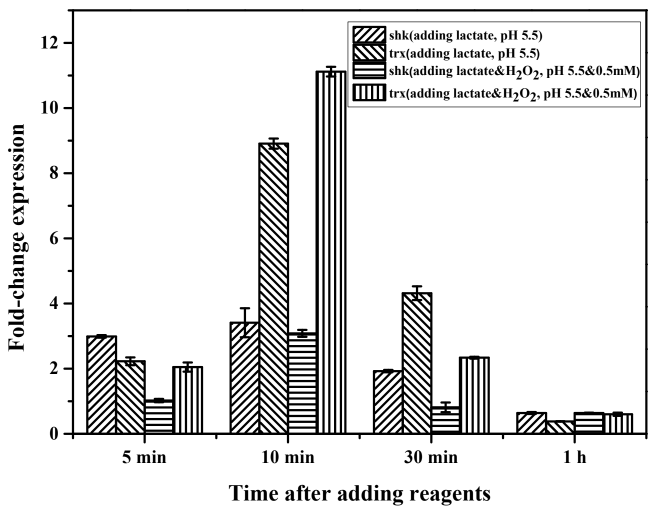

Supplement: Supplementary file 1 [file microorganisms-11-00695-s001.zip › microorganisms-2263676-supplementary.pdf]
